# New records and an updated checklist of snakes from Son La Province, Vietnam

**DOI:** 10.3897/BDJ.8.e52779

**Published:** 2020-06-17

**Authors:** Anh Van Pham, Thomas Ziegler, Truong Quang Nguyen

**Affiliations:** 1 Tay Bac University, Quyet Tam Ward, Son La City, Son La Province, Vietnam Tay Bac University, Quyet Tam Ward, Son La City Son La Province Vietnam; 2 AG Zoologischer Garten Köln, Riehler Straße 173, D-50735, Cologne, Germany AG Zoologischer Garten Köln, Riehler Straße 173, D-50735 Cologne Germany; 3 Institute of Zoology, University of Cologne, Zülpicher Street 47b, D–50674, Cologne, Germany Institute of Zoology, University of Cologne, Zülpicher Street 47b, D–50674 Cologne Germany; 4 Institute of Ecology and Biological Resources, Vietnam Academy of Science and Technology, 18 Hoang Quoc Viet Road, Hanoi, Vietnam Institute of Ecology and Biological Resources, Vietnam Academy of Science and Technology, 18 Hoang Quoc Viet Road Hanoi Vietnam; 5 Graduate University of Science and Technology, Vietnam Academy of Science and Technology, 18 Hoang Quoc Viet Road, Hanoi, Vietnam Graduate University of Science and Technology, Vietnam Academy of Science and Technology, 18 Hoang Quoc Viet Road Hanoi Vietnam

**Keywords:** Distribution, new records, morphology, taxonomy

## Abstract

**Background:**

Son La Province is located in north-western Vietnam and the province contains a large area of 440,000 hectares of natural forest. A wide range of elevations and the complexity of landforms have given this province a great diversity of natural habitats and a high level of biodiversity potential. However, the snake fauna of Son La province is still poorly studied. Previous studies documented 56 species of snakes from this province.

**New information:**

As a result of our field surveys in Phu Yen, Song Ma, Thuan Chau and Van Ho districts, we report five species of snakes for the first time from Son La Province, northern Vietnam, namely *Boiga cyanea, B. guangxiensis*, *Lycodon meridionalis* (Colubridae), *Protobothrops cornutus* and *P. mucrosquamatus* (Viperidae), with novel data about morphological characters. In addition, we provide an updated checklist of 61 snake species from Son La Province. The snake fauna of Son La Province also contains a number of species of conservation concern: 11 species listed in the Red Data Book of Vietnam (2007), four species listed in the IUCN Red List (2020) and four species listed in the Vietnam Governmental Decree No. 06 (2019).

## Introduction

Son La Province is located in north-western Vietnam with an area of 14,118.9 km^2^. The province contains a large area of 440,000 hectares of natural forest (The People's Committee of Son La Province 2007). A wide range of elevations (up to 2979 m a.s.l.) and the complexity of landforms have given this province a great diversity of natural habitats and a high level of biodiversity potential. However, the snake fauna of this province is still poorly studied. In their herpetofaunal list of Vietnam, Nguyen et al. (2009) listed 42 species of snakes from the province. Pham et al. (2013)and Pham et al. (2014) documented 11 new provincial records of snakes, based on newly-collected voucher specimens from Son La Province. Since then, several new discoveries of snakes have been reported from this province. In 2015, two snake species were reported for the first time from Vietnam: *Sinonatrix yunnanensis* Rao & Yang (Le et al. 2015) from Sop Cop Nature Reserve and *Parafimbrios lao* Teynié, David, Lottier, Le, Vidal & Nguyen (Nguyen et al. 2015) from Copia Nature Reserve, two protected areas located in the western part of Son La Province. Remarkably, a new species of odd-scaled snake was recently described from Copia Nature Reserve in Son La Province, namely *Achalinus timi* Ziegler, Nguyen, Pham, Nguyen, Pham, Van Schingen, Nguyen & Le (Ziegler et al. 2019).

Based on our recent fieldwork in Son La Province between 2015 and 2017, we herein provide five new records and an updated checklist of snakes from Son La Province.

## Materials and methods

**Figure 1.**
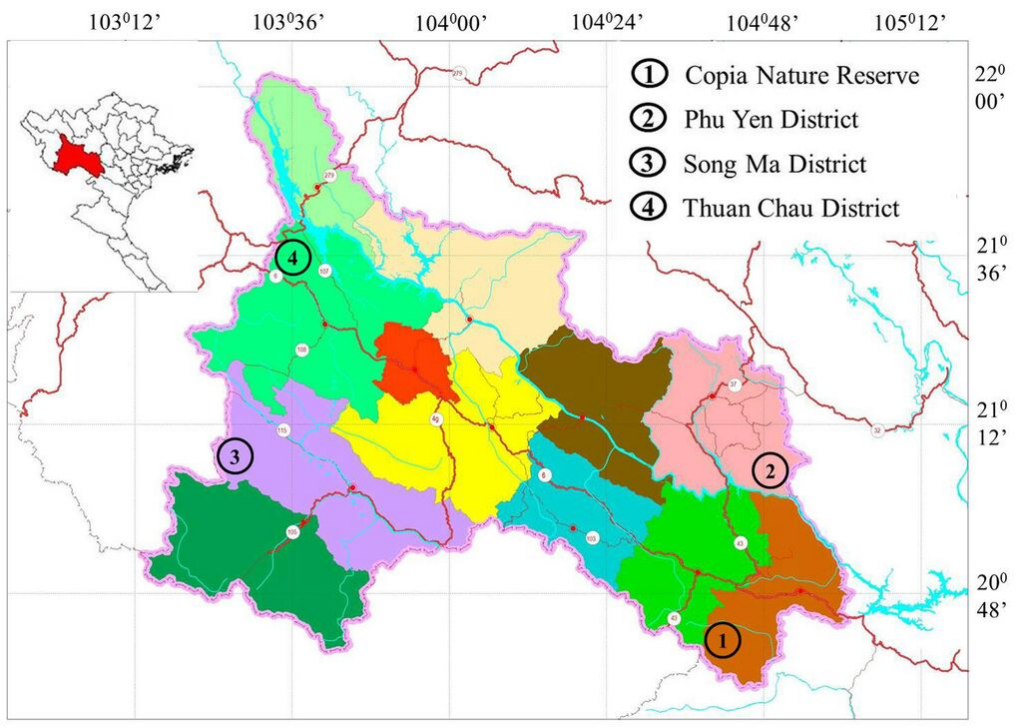
Map showing the survey sites in Son La Province, north-western Vietnam

**Sampling.** Four field surveys were conducted in Son La Province, Vietnam: from 28 August to 4 September 2015 in the Phu Yen District; from 24 to 28 June 2016 in the Xuan Nha Nature Reserve of Van Ho District; from 24 to 26 September 2016 in the Thuan Chau District; and from 15 to 19 April 2017 in the Song Ma District (Fig. 1). The coordinates were recorded by using a Garmin GPSmap 60Cx (WGS 84 datum).

Snakes were collected by hand between 08:00 and 22:00 h. After taking photographs in life, snake individuals were euthanised in a closed vessel with a piece of cotton wool containing ethyl acetate (Simmons 2002), fixed in 85% ethanol and then transferred to 70% ethanol for permanent storage. Road-killed snakes were also collected for morphological analysis. Voucher specimens were subsequently deposited in the collections of the Tay Bac University (TBU), Son La Province, Vietnam.

**Morphological characters.** Measurements, except body and tail lengths, were taken with a slide-calliper to the nearest 0.1 mm in preserved specimens; all measures on body were measured to the nearest millimetre. The number of ventral scales was counted according to Dowling (1951). The number of dorsal scale rows were counted at one head length behind head, at midbody (i.e. at the level of the ventral plate corresponding to half of the total ventral number) and at one head length before vent, respectively. The terminal, pointed scute was not included in the number of subcaudals. Values for symmetric head characters were given in left/right order. The following abbreviations were used: SVL: Snout-vent length, TaL: Tail length.

## Taxon treatments

### Boiga cyanea

(Duméril, Bibron & Duméril, 1854)

747FC998-B231-57E0-BE36-42A30B749F77

#### Materials

- **Type status:** Other material. **Occurrence:** catalogNumber: TBU.2017. 01; individualCount: 1; sex: female; lifeStage: adult; **Taxon:** scientificNameID: Boiga cyanea; scientificName: Boiga cyanea; class: Reptilia; order: Squamata; family: Colubridae; genus: Boiga; specificEpithet: cyanea; scientificNameAuthorship: (Duméril, Bibron & Duméril, 1854); **Location:** country: Vietnam; countryCode: VN; stateProvince: Son La; county: Song Ma; municipality: Pu Bau; locality: near Pa Lau Village; verbatimElevation: 590 m; verbatimLatitude: 21°15'07''N; verbatimLongitude: 103°26'04''E; verbatimCoordinateSystem: WGS84; **Event:** eventDate: 16 April 2017; eventRemarks: collected by N.B. Sung; **Record Level:** language: en; collectionCode: Reptilia; basisOfRecord: PreservedSpecimen

#### Description

Morphological characters of the voucher specimen from Son La Province agreed well with the descriptions of Smith (1943), Tillack (2006) and Ziegler et al. (2010): SVL 1100 mm; TaL 380 mm (n = 1); head distinct from neck; eye large, pupil round; rostral broader than high; internasal not in contact with loreal; prefrontal longer than half of frontal; frontal pentagonal; parietals longer than wide; nasal divided; loreal 1/1, not in contact with orbit; preocular 1/1; postoculars 2/2, bordering anterior temporals; anterior temporals 2/2, posterior temporals 3/3; subpralabials 8/8, third to fifth touching the eye, seventh largest; infralabials 11/11, first to fifth bordering chin shields; dorsal scale rows 23-21-15, smooth; ventrals 242; cloacal scale undivided; subcaudals 128, paired.

**Figure 2.**
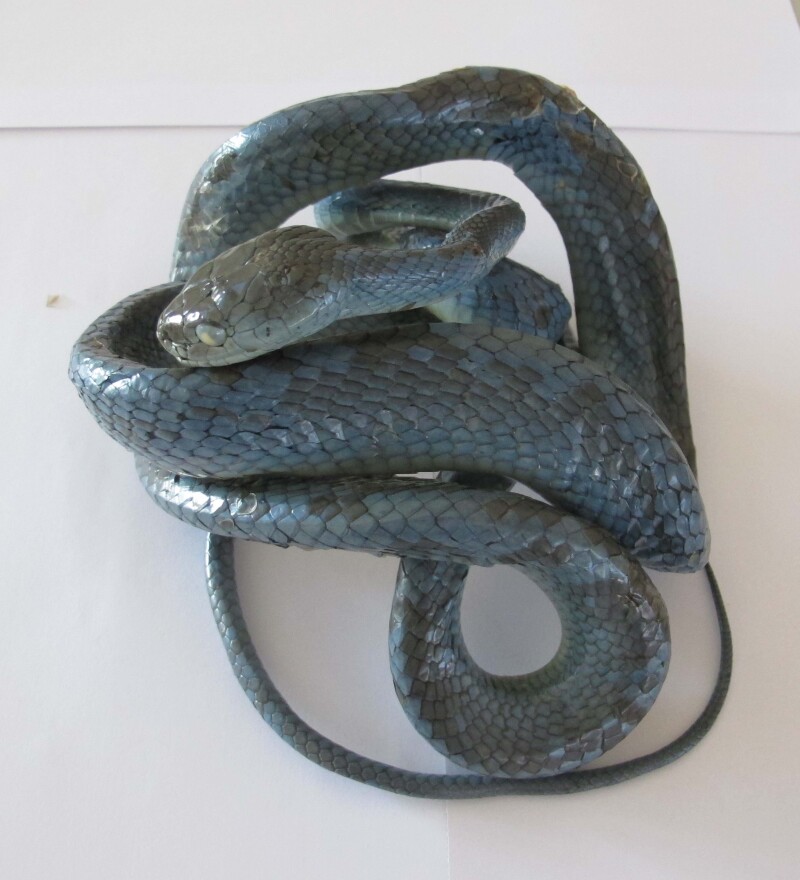
*Boiga cyanea* (TBU 2017.01, adult female) from Son La Province, Vietnam. Photo: A.V. Pham.

Colouration in preservative: dorsal surface greenish-grey; belly whitish-grey (Fig. 2).

#### Distribution

In Vietnam, this species has been recorded from Lao Cai Province in the north, southwards to Ho Chi Minh City (Nguyen et al. 2009, Ziegler et al. 2010). Elsewhere, this species is known from India, Bangladesh, Bhutan, Nepal, China, Myanmar, Laos, Thailand, Cambodia and Malaysia (Nguyen et al. 2009, Ziegler et al. 2010).

#### Ecology

The voucher specimen was collected at 19:30 h on the ground. The surrounding habitat was secondary forest, composed of small hardwoods, lianes and shrub. Air temperature was 25-30^o^C and relative humidity was 70-75%.

### Boiga guangxiensis

Wen, 1998

C7B250B1-A988-5CAB-B81C-88C2311380E8

#### Materials

- **Type status:** Other material. **Occurrence:** catalogNumber: TBU. SL.2016.128; individualCount: 1; sex: female; lifeStage: adult; **Taxon:** scientificNameID: Boiga guangxiensis; scientificName: Boiga guangxiensis; class: Reptilia; order: Squamata; family: Colubridae; genus: Boiga; specificEpithet: guangxiensis; scientificNameAuthorship: Wen, 1998; **Location:** country: Vietnam; countryCode: VN; stateProvince: Son La; county: Van Ho; municipality: Chieng Xuan; locality: near Kho Hong Village; verbatimElevation: 1090 m; verbatimLatitude: 21°43'18''N; verbatimLongitude: 104°40'26''E; verbatimCoordinateSystem: WGS84; **Event:** eventDate: 26 June 2016; eventRemarks: collected by A.V. Pham and N.B. Sung; **Record Level:** language: en; collectionCode: Reptilia; basisOfRecord: PreservedSpecimen

#### Description

Morphological characters of the voucher specimen from Son La Province agreed well with the descriptions of Das (2010) and Ziegler et al. (2010): SVL 1395 mm, tail tip lost, TaL 161 mm (n = 1); head distinct from neck; eye large, pupil round; rostral broader than high; internasal not in contact with loreal; prefrontal longer than half of frontal; frontal pentagonal; parietals longer than wide; nasal divided; loreal 1/1, not in contact with orbit; preocular 1/1; postoculars 2/2, bordering anterior temporals; anterior temporals 2/2, posterior temporals 3/3; supralabials 8/9, third to fifth touching the eye, seventh largest; infralabials 12/12, first to third bordering chin shields; dorsal scale rows 23-21-15, smooth; ventrals 262; cloacal scale undivided; subcaudals 55+, paired.

**Figure 3.**
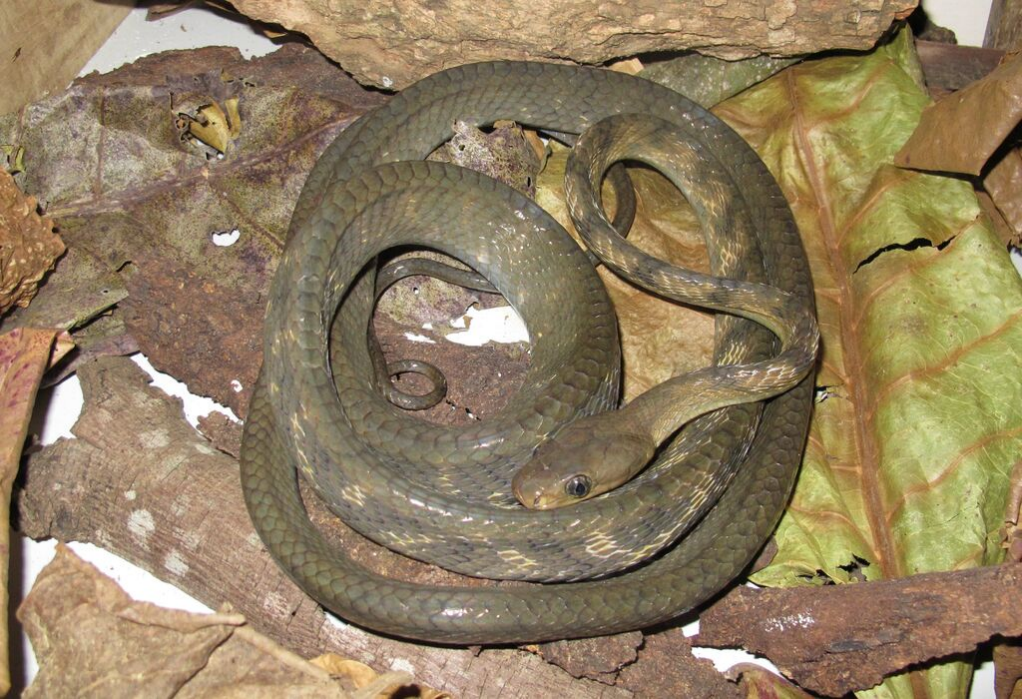
*Boiga guangxiensis* (TBU SL.2016. 128, adult female) from Son La Province, Vietnam. Photo: A.V. Pham

Colouration in life: dorsal surface grey, with dark spots on the anterior body; dorsolateral side light brown on anterior body; belly whitish anteriorly, grey posteriorly (Fig. 3).

#### Distribution

In Vietnam, this species has been recorded from Lai Chau and Lao Cai provinces in the north, southwards to Kon Tum and Lam Dong provinces (Nguyen et al. 2009). Elsewhere, this species is known from China and Laos (Nguyen et al. 2009).

#### Ecology

The voucher specimen was collected at 20:50 h on a tree branch, approximately 1.0 m above the ground. The surrounding habitat was disturbed evergreen karst forest of medium hardwood and shrub. Air temperature was 25-30°C and relative humidity was 70-75%.

### Lycodon meridionalis

(Bourret, 1935)

C3F74650-FA9D-5108-AF7C-59D21FC2E53D

#### Materials

- **Type status:** Other material. **Occurrence:** catalogNumber: TBU. SL.2016.144; individualCount: 1; sex: female; lifeStage: adult; **Taxon:** scientificNameID: Lycodon meridionalis; scientificName: Lycodon meridionalis; class: Reptilia; order: Squamata; family: Colubridae; genus: Lycodon; specificEpithet: meridionalis; scientificNameAuthorship: (Bourret, 1935); **Location:** country: Vietnam; countryCode: VN; stateProvince: Son La; county: Van Ho; municipality: Chieng Xuan; locality: near Hua Lat Mountain; verbatimElevation: 1080 m; verbatimLatitude: 20°46'84''N; verbatimLongitude: 104°49'00''E; verbatimCoordinateSystem: WGS84; **Event:** eventDate: 28 June 2016; eventRemarks: collected by A.V. Pham and N.B. Sung; **Record Level:** language: en; collectionCode: Reptilia; basisOfRecord: PreservedSpecimen

#### Description

Morphological characters of the voucher specimen from Son La Province agreed well with the descriptions of Orlov and Ryabov (2004), Hecht et al. (2013), Nguyen et al. (2016) and Nguyen et al. (2018): SVL 810 mm, TaL 227 mm (n = 1); head distinct from neck; internasals not in contact with loreal; nasal divided; loreal 1/1, not touching the eye; preocular 1/1; subocular absent; postoculars 2/2; anterior temporals 2/3, posterior temporals 3/3; supralabials 8/8, third to fifth entering orbit; infralabials 10/10; dorsal scale rows 17-17-15, strongly keeled except 5 outermost rows smooth, outer dorsal scales enlarged; ventrals 250 (+ 2 preventrals); cloacal undivided; subcaudals 104, divided.

**Figure 4.**
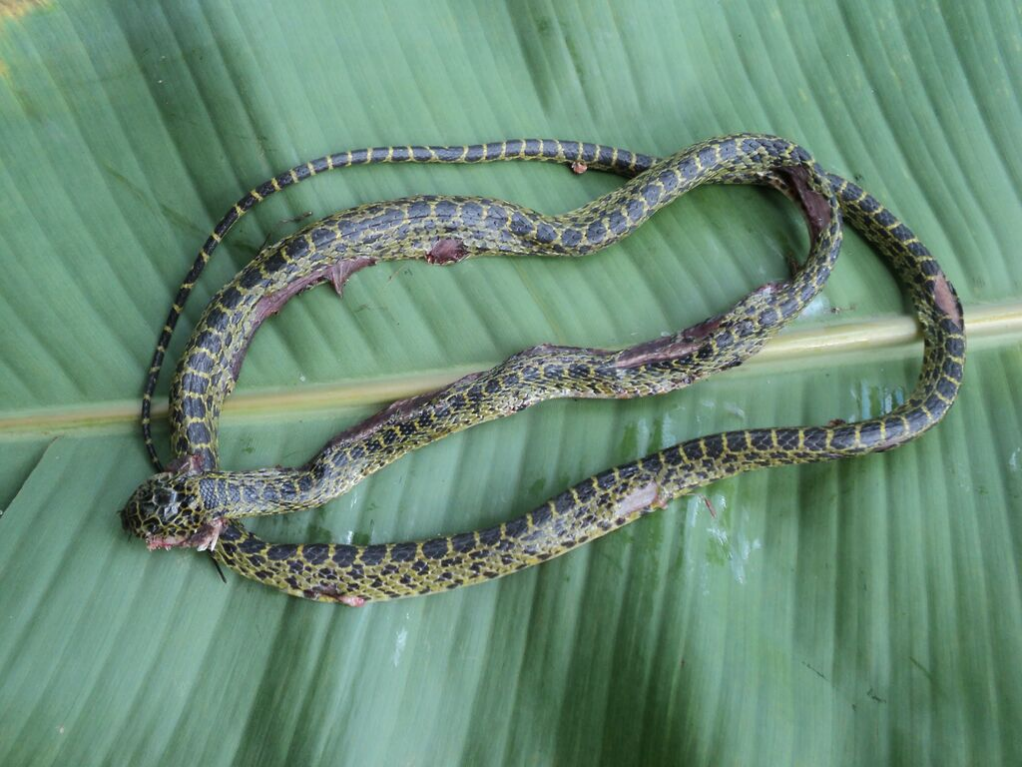
*Lycodon meridionalis* (TBU SL.2016. 144, adult female) from Son La Province, Vietnam. Photo: N.B. Sung

Colouration in life: dorsal surface black, with 122 narrow yellow cross-bars on body and 35 on tail, bifurcated on the sides, enclosing dark spots; head dark black with symmetrical light markings, the most conspicuous being the one running from the eye to the margin of the snout and another stretching from the hind margin of the parietals; belly cream; lower surface of tail dark brown (Fig. 4).

#### Distribution

In Vietnam, this species has been reported from Lao Cai and Ha Giang provinces in the north, southwards to Thanh Hoa Province (Nguyen et al. 2009, Ziegler et al. 2014, Nguyen et al. 2018, Nguyen et al. 2016, Nguyen et al. 2018). Elsewhere, the species has been reported from China and Laos (Nguyen et al. 2009, Ziegler et al. 2014, Nguyen et al. 2016, Nguyen et al. 2018).

#### Ecology

The voucher specimen was found at 09:10 h on the ground. The surrounding habitat was secondary forest composed of medium and small hardwoods, lianes and shrub. Air temperature was 27-32°C and relative humidity was 65-70%.

### Protobothrops cornutus

(Smith, 1930)

EE026855-1034-5FCB-9417-352EA84D5C56

#### Materials

- **Type status:** Other material. **Occurrence:** catalogNumber: TBU. MD.2015.110; individualCount: 1; sex: male; lifeStage: adult; **Taxon:** scientificNameID: Protobothrops cornutus; scientificName: Protobothrops cornutus; class: Reptilia; order: Squamata; family: Viperidae; genus: Protobothrops; specificEpithet: cornutus; scientificNameAuthorship: (Smith, 1930); **Location:** country: Vietnam; countryCode: VN; stateProvince: Son La; county: Phu Yen; municipality: Muong Do; locality: near Pap Village; verbatimElevation: 720 m; verbatimLatitude: 21°12'36''N; verbatimLongitude: 104°45'58''E; verbatimCoordinateSystem: WGS84; **Event:** eventDate: 26 August 2015; eventRemarks: collected by H.V. Tu and C.A.K.P Kham; **Record Level:** language: en; collectionCode: Reptilia; basisOfRecord: PreservedSpecimen

#### Description

Morphological characters of the voucher specimen from Son La Province agreed well with the description of Herrmann et al. (2004) and Luu et al. (2015): SVL 610 mm, TaL 146 mm (n = 1); body elongated; head triangular, distinct from neck, flat, covered by small scales; snout obtuse; rostral scale triangular, not visible above; nasal single, subrectangular; loreals 3/3, between nasal and preocular scale; loreal pit bordered by second supralabial and two preocular scales; supralabials 10/10, first completely separated from nasal, third supralabial largest, concave in the middle; horns including one enlarged scale and two relatively-enlarged supraoculars; supralabials separated from suboculars by 2 scales; infralabials 13/12, the first pair in contact with each other, the first three pairs in contact with the chin shields; dorsal scale rows 21-21-17, keeled; ventral scales 190; subcaudal scales 71, all paired; cloacal entire.

**Figure 5.**
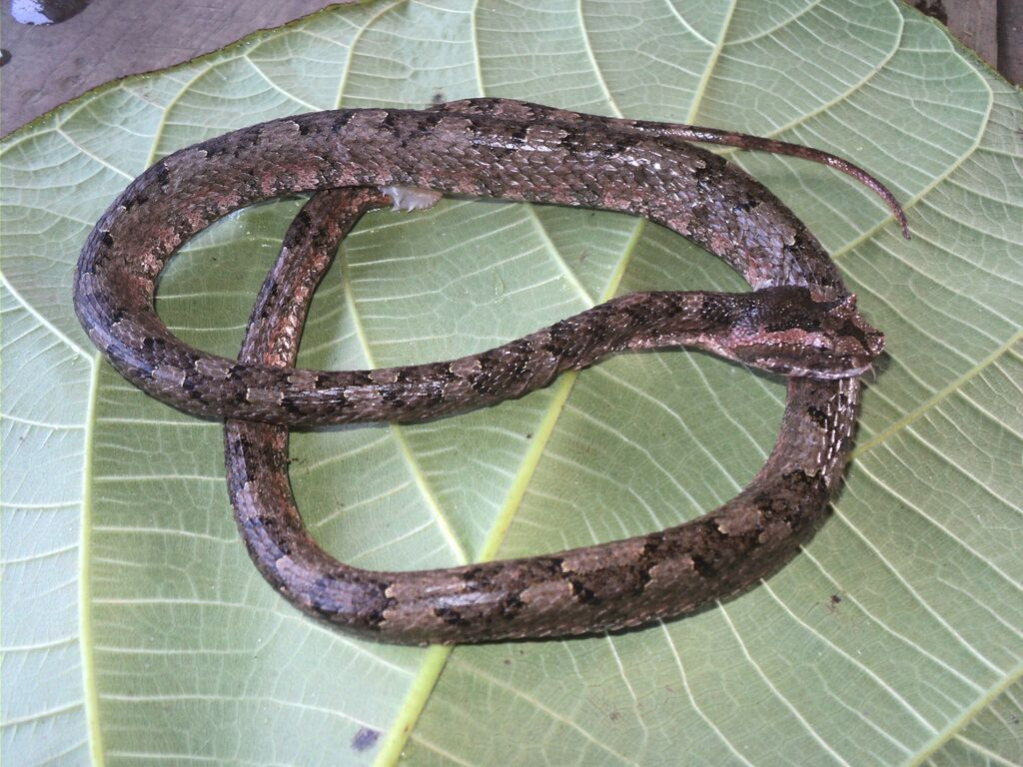
*Protobothrops cornutus* (TBU MD.2015.110, adult male) from Son La Province, Vietnam. Photo: H.V. Tu

Colouration in life: dorsal head grey with dark brown marks; one stripe each side from posterior orbit to edge of the labial; dorsal surface grey-brown with serial blotches along the midbody; dorsolateral side light brown; ventral surface greyish-blue (Fig. 5).

#### Distribution

In Vietnam, this species has been reported from Lai Chau and Lao Cai provinces in the north, southwards to Quang Binh and Thua Thien Hue provinces (Nguyen et al. 2009). Elsewhere, the species has been reported from China (Nguyen et al. 2009).

#### Ecology

The voucher specimen was found at 20:35 h on the ground. The surrounding habitat was secondary forest composed of medium and small hardwoods, lianes and shrub. Air temperature was 25-30°C and relative humidity was 70-80%.

### Protobothrops mucrosquamatus

(Cantor, 1839)

63E7EECA-0195-5D9A-9F6B-49A2230DB1CC

#### Materials

- **Type status:** Other material. **Occurrence:** catalogNumber: TBU. SL.2016.423; individualCount: 1; sex: male; lifeStage: adult; **Taxon:** scientificNameID: Protobothrops mucrosquamatus; scientificName: Protobothrops mucrosquamatus; class: Reptilia; order: Squamata; family: Viperidae; genus: Protobothrops; specificEpithet: mucrosquamatus; scientificNameAuthorship: (Cantor, 1839); **Location:** country: Vietnam; countryCode: VN; stateProvince: Son La; county: Phu Yen; municipality: Muong Bang; locality: near Cai Village; verbatimElevation: 450 m; verbatimLatitude: 21°08'49''N; verbatimLongitude: 104°44'48''E; verbatimCoordinateSystem: WGS84; **Event:** eventDate: 26 October 2016; eventRemarks: collected by A.V. Pham and S.V. Cao; **Record Level:** language: en; collectionCode: Reptilia; basisOfRecord: PreservedSpecimen
- **Type status:** Other material. **Occurrence:** catalogNumber: TBU. PL.2016.67; individualCount: 1; sex: female; lifeStage: adult; **Taxon:** scientificNameID: Protobothrops mucrosquamatus; scientificName: Protobothrops mucrosquamatus; class: Reptilia; order: Squamata; family: Viperidae; genus: Protobothrops; specificEpithet: mucrosquamatus; scientificNameAuthorship: (Cantor, 1839); **Location:** country: Vietnam; countryCode: VN; stateProvince: Son La; county: Thuan Chau; municipality: Muong Bang; locality: near Pha Khuong Village; verbatimElevation: 720 m; verbatimLatitude: 21°36'32''N; verbatimLongitude: 103°34'54''E; verbatimCoordinateSystem: WGS84; **Event:** eventDate: 25 September 2016; eventRemarks: collected by A.V. Pham T.Q.L. Hoang, L.M. Ha, N.B. Song, D.K.K.S. Vanh, and C.A. Lau; **Record Level:** language: en; collectionCode: Reptilia; basisOfRecord: PreservedSpecimen

#### Description

Morphological characters of the voucher specimens from Son La agreed well with the descriptions of Stuart and Heatwole (2008), Nguyen et al. (2011), Luu et al. (2013), Nemes et al. (2013) and Nguyen et al. (2018): SVL 870 and 950 mm, TaL 220 and 235 mm (n = 2); head triangular, clearly distinct from the neck; nasal undivided; internasals separated from each other by three scales; singe loreal pit; two small scales between the nasal and the shield bordering the anterior region of the loreal pit; postoculars 2/2; supralabials 9-11/9-11, the third large, fourth and fifth separated from the subocular by two scales; temporals small; infralabials 12-13/13-14, the first pair in contact with each other, the first three pairs in contact with the chin shields; dorsal scale rows 27-25-19, rhomboid, strongly keeled throughout, but smooth on the first outer row; ventrals 212-217 (+2 preventrals); cloacal undivided; subcaudals 94 and 99, divided.

**Figure 6.**
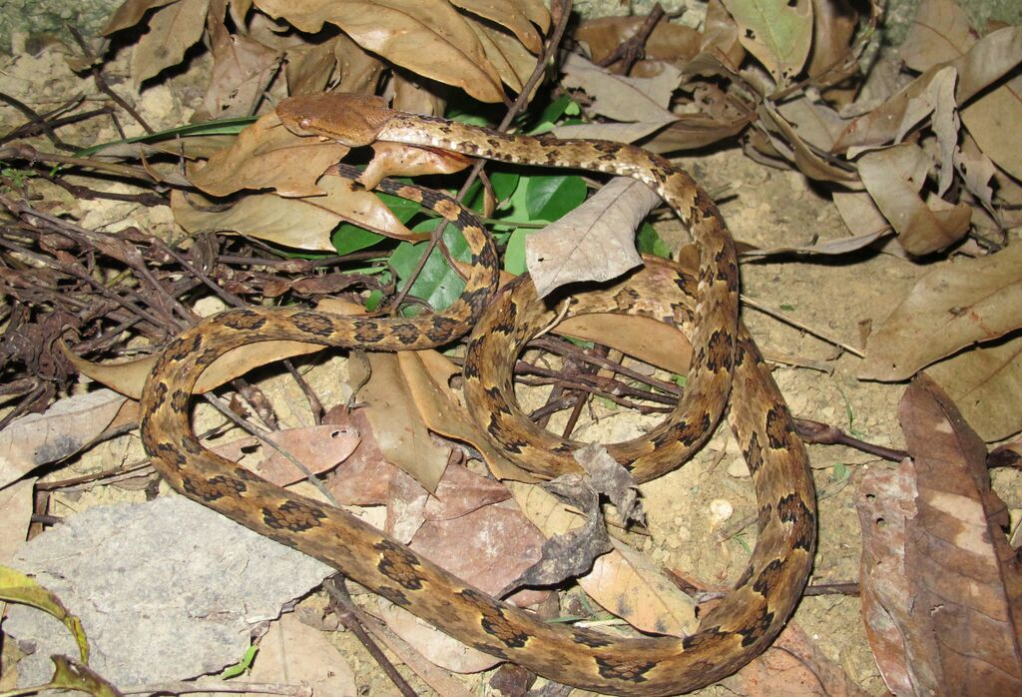
*Protobothrops mucrosquamatus* (TBU PL.2016. 67, adult male) from Son La Province, Vietnam. Photo: A. V. Pham

Colouration in life: dorsal head brown, paler below; dorsum greyish-brown, with a series of large brown, dark-edged spots; a dark brown line from the eye to the angle of the mouth, edged in black; ventral surface brownish with white blotches; dorsal tail light brown, with a series of conspicuous black spots (Fig. 6).

#### Distribution

In Vietnam, this species has been reported from Lao Cai and Ha Giang provinces in the north, southwards to Kon Tum and Gia Lai provinces (Nguyen et al. 2009, Nemes et al. 2013). Elsewhere, the species has been reported from India, Bangladesh, China, Taiwan and Myanmar (Nguyen et al. 2009, Nemes et al. 2013).

#### Ecology

The voucher specimens were found between 19:00 and 22:30 h on forest paths. The surrounding habitat was secondary forest composed of medium and small hardwoods, lianes and shrub. Air temperature was 22-27°C and relative humidity was 75-85%.

## Discussion

Amongst four survey sites in Son La Province, the snake species number recorded in Thuan Chau District was highest (47), followed by Phu Yen District (35 species), Song Ma District (33 species) and Van Ho District (29 species). The forest area in Thuan Chau District (63,260 ha) is larger and the habitat quality is better than those in other districts (forest area of Phu Yen District: 59,493 ha, Song Ma District: 55,814 ha and Van Ho District: 51,528 ha, respectively) (The People's Committee of Son La Province 2007). Our new findings bring the species number of snakes to 61 in Son La Province. The most diverse family is Colubridae with 28 recorded species, followed by Natricidae (10 species), Elapidae (6 species), Viperidae (5 species), Pareatidae (3 species), Xenodermidae and Homalopsidae (2 species), Lamprophiidae, Pseudoxenodontidae, Pythonidae, Typhlopidae and Xenopeltidae (1 species each).

In terms of conservation concern, Son La Province also harbours a high number of threatened snake species: 11 species listed in the Red Data Book of Vietnam (Dang et al. 2007): *Python molurus*, *Coelognathus radiatus*, *Euprepiophis mandarinus*, *Gonyosoma prasinus*, *Oreocryptophis porphyraceus*, *Elaphe moellendorffi*, *Ptyas korros*, *Ptyas mucosa*, *Bungarus fasciatus*, *Naja atra* and *Ophiophagus hannah*; four species listed in the IUCN Red List (IUCN 2020): *Python molurus, Elaphe moellendorffi*, *Ophiophagus hannah* and *Protobothrops cornutus*; and four species listed in the Vietnam Governmental Decree No. 06/2019/ND-CP (The Government of Vietnam 2019): *Python molurus*, *Ptyas mucosa*, *Naja atra* and *Ophiophagus hannah* (see Table 1).

## Supplementary Material

XML Treatment for Boiga cyanea

XML Treatment for Boiga guangxiensis

XML Treatment for Lycodon meridionalis

XML Treatment for Protobothrops cornutus

XML Treatment for Protobothrops mucrosquamatus

## Figures and Tables

**Table 1.** List of snake species recorded from Son La Province, Vietnam [Decree 06 (2019) = The Governmental Decree No 06/2019/ND-CP, dated 22 January 2019 by the Government of Vietnam on the management of endangered wild flora and fauna. Group IB: prohibited exploitation and use for commercial purpose, Group IIB: limited exploitation and use for commercial purpose; RBVN (2007) = Vietnam Red Data Book (Dang et al. 2007). IUCN (2020) = The IUCN Red List of Threatened Species. CR = Critically Endangered, EN = Endangered, VU = Vulnerable, NT = Lower Risk/Near Threatened]

**Name**	**Previous record**	**RBVN 2007**	**IUCN 2020**	**Decree 06** **2019**
** Typhlopidae **				
*Indotyphlops braminus* (Daudin, 1803)	Nguyen et al. 2009			
** Pythonidae **				
*Python molurus* (Linnaeus, 1758)	Nguyen et al. 2009	CR	VU	IIB
** Xenopeltidae **				
*Xenopeltis unicolor* Reinwardt, 1827	Nguyen et al. 2009			
** Colubridae **				
*Calamaria pavimentata* Dumeril, Bibron & Dumerin, 1854	Nguyen et al. 2009			
*Ahaetulla prasina* (Boie, 1827)	Nguyen et al. 2009			
*Boiga cyanea* (Dumeril, Bibron & Dumeril, 1854)	This study			
*Boiga guangxiensis* Wen, 1998	This study			
*Boiga multomaculata* (Boie, 1827)	Nguyen et al. 2009			
*Chrysopelea ornata* (Shaw, 1802)	Nguyen et al. 2009			
*Coelognathus radiatus* (Boie, 1827)	Nguyen et al. 2009	EN		
*Dendrelaphis ngansonensis* (Bourret, 1935)	Nguyen et al. 2009			
*Dendrelaphis pictus* (Gmelin, 1789)	Pham et al. 2013			
*Euprepiophis mandarinus* (Cantor, 1842)	Nguyen et al. 2009	VU		
*Gonyosoma frenatum* (Gray, 1853)	Pham et al. 2013			
*Gonyosoma prasinus* (Blyth, 1854)	Pham et al. 2014	VU		
*Liopeltis frenatus* (Gunther, 1858)	Pham et al. 2013			
*Lycodon fasciatus* (Anderson, 1879)	Nguyen et al. 2009			
*Lycodon futsingensis* (Pope, 1928)	Nguyen et al. 2009			
*Lycodon meridionalis* (Bourret, 1935)	This study			
*Lycodon subcinctus* Boie, 1827	Nguyen et al. 2009			
*Oligodon catenatus* (Blyth, 1854)	Pham et al. 2014			
*Oligodon chinensis* (Gunther, 1888)	Pham et al. 2014			
*Oligodon fasciolatus* (Günther, 1864)	Nguyen et al. 2009			
*Oreocryptophis porphyraceus* (Cantor, 1839)	Nguyen et al. 2009	VU		
*Elaphe moellendorffi* (Boettger, 1886)	Nguyen et al. 2009	VU	VU	
*Orthriophis taeniurus* (Cope, 1861)	Nguyen et al. 2009			
*Ptyas korros* (Schlegel, 1837)	Nguyen et al. 2009	EN		
*Ptyas multicinctus* (Roux, 1907)	Pham et al. 2014			
*Ptyas mucosa* (Linnaeus, 1758)	Nguyen et al. 2009	EN		IIB
*Gonyosoma boulengeri* Mocquard, 1897	Nguyen et al. 2009			
*Sibynophis collaris* (Gray, 1853)	Nguyen et al. 2009			
** Elapidae **				
*Bungarus fasciatus* (Schneider, 1801)	Nguyen et al. 2009	EN		
*Bungarus multicinctus* Blyth, 1860	Nguyen et al. 2009			
*Naja atra* Cantor, 1842	Nguyen et al. 2009	EN		IIB
*Ophiophagus hannah* (Cantor, 1836)	Nguyen et al. 2009	CR	VU	IB
*Sinomicrurus kelloggi* (Pope, 1928)	Nguyen et al. 2009			
*Sinomicrurus macclellandi* (Reinhardt, 1844)	Nguyen et al. 2009			
** Homalopsidae **				
*Hypsiscopus plumbea* (Boie, 1827)	Nguyen et al. 2009			
*Myrrophis chinensis* (Gray, 1842)	Nguyen et al. 2009			
** Lamprophiidae **				
*Psammodynastes pulverulentus* (Boie, 1827)	Nguyen et al. 2009			
** Natricidae **				
*Amphiesma stolatum* (Linnaeus, 1758)	Nguyen et al. 2009			
*Hebius bitaeniatum* (Wall, 1925)	Nguyen et al. 2009			
*Hebius boulengeri* (Gressitt, 1937)	Pham et al. 2013			
*Hebius chapaensis* Bourret, 1934	Pham et al. 2013			
*Rhabdophis chrysargos* (Schlegel, 1837)	Nguyen et al. 2009			
*Rhabdophis nigrocinctus* (Blyth, 1856)	Nguyen et al. 2009			
*Rhabdophis subminiatus* (Schlegel, 1837)	Nguyen et al. 2009			
*Sinonatrix percarinata* (Boulenger, 1899)	Nguyen et al. 2009			
*Sinonatrix yunnanensis* Rao & Yang, 1998	Le et al. 2015			
*Fowlea flavipunctatus* (Hallwell, 1861)	Nguyen et al. 2009			
** Pareatidae **				
*Pareas carinatus* (Boie, 1828)	Nguyen et al. 2009			
*Pareas hamptoni* (Boulenger, 1905)	Pham et al. 2013			
*Pareas margaritophorus* (Jan, 1866)	Pham et al. 2013			
** Pseudoxenodontidae **				
*Pseudoxenodon macrops* (Blyth, 1854)	Pham et al. 2014			
** Viperidae **				
*Ovophis monticola* (Günther, 1864)	Pham et al. 2013			
*Protobothrops cornutus* (Smith, 1930)	This study		NT	
*Protobothrops mucrosquamatus* (Cantor, 1839)	This study			
*Trimeresurus albolabris* Gray, 1842	Nguyen et al. 2009			
*Trimeresurus stejnegeri* Schmidt, 1925	Nguyen et al. 2009			
** Xenodermidae **				
*Achalinus timi* Ziegler, Nguyen, Pham, Nguyen, Pham, Van Schingen, Nguyen & Le, 2019	Ziegler et al. 2019			
*Parafimbrios lao* Teynié, David, Lottier, Le, Vidal & Nguyen, 2015	Nguyen et al. 2015			
